# Flipped Classroom Formats in a Problem-Based Learning Course: Experiences of First-Year Bachelor European Public Health Students

**DOI:** 10.3389/phrs.2022.1604795

**Published:** 2022-08-04

**Authors:** Nynke de Jong, Peter van Rosmalen, Maria Teresa Brancaccio, Michel H. C. Bleijlevens, Hilde Verbeek, Inge G. P. Peeters

**Affiliations:** ^1^ Department of Health Services Research, Faculty of Health, Medicine and Life Sciences, Maastricht University, Maastricht, Netherlands; ^2^ School of Health Professions Education, Faculty of Health, Medicine and Life Sciences, Maastricht University, Maastricht, Netherlands; ^3^ Department of Educational Development and Research, Faculty of Health, Medicine and Life Sciences, Maastricht University, Maastricht, Netherlands; ^4^ Department of Health, Ethics, and Society, Faculty of Health, Medicine and Life Sciences, Maastricht University, Maastricht, Netherlands; ^5^ Care and Public Health Research Institute, Faculty of Health, Medicine and Life Sciences, Maastricht University, Maastricht, Netherlands; ^6^ Living Lab in Ageing and Long-Term Care, Maastricht, Netherlands; ^7^ Department of Family Medicine & Academy for Postgraduate Medical Training, Maastricht University | Maastricht UMC+ Academy, Maastricht, Netherlands

**Keywords:** problem-based learning, flipped classroom, education in ageing in Europe, bachelor European Public Health students, expert meetings in education

## Abstract

**Objectives:** Students would like to see more creativity and flexibility in the performance of problem-based learning (PBL). Therefore, we applied flipped classroom formats in a course of the Bachelor European Public Health at Maastricht University to investigate the experiences of the students. The main objective was to stimulate interaction between students mutual, and between students and teachers.

**Methods:** 304 first-year students following the course on “Ageing in Europe” in three academic years, were asked to fill out questions focussing on prior knowledge, preparation work, and group session parameters, e.g., duration, content, extent of interaction and format group session.

**Results:** In-class activities, such as debate, making a mind map, giving a pitch, role-play e.g., were highly appreciated by students, especially the interactivity and discussions with the experts during these sessions. Students felt they applied knowledge.

**Conclusion:** Flipped classroom formats can be used to extend the Maastricht University PBL design and students do recommend this. It can be a relevant and challenging answer on the articulated request for more creativity and flexibility in the regular PBL format.

## Introduction

All over the world, higher education institutes embrace problem-based learning (PBL) approaches [1]. The reason for this is that this educational method encourage students in their learning, “*to get students to adopt a deep approach to studying*” [2], p. 880. The four underlying key learning principles behind PBL are, that learning should be constructive-, self-regulated, collaborative and contextual [3]. These learning principles should enhance deep learning. A review of the literature in PBL, in which twenty-one studies were included, seems to confirm this statement by concluding that PBL seems to enhance deep learning [4].

At Maastricht University, PBL has been at the heart of the university ever since the university was founded (i.e., 1974) and while still successful, there is a need to consider ongoing developments. New methods have been developed since and might be beneficial to strengthen the current PBL implementation. The PBL-approach is aimed at achieving two educational objectives: the acquisition of an integrated body of knowledge related to problems and the development or application of problem-solving skills [5]. As a student, you work in small groups, engage in hands-on training and attend (far) fewer lectures than in traditional education. Under the supervision of a tutor, you team up with ten to twelve students to tackle real-life challenges, in so-called tutorials. In classic face-to-face tutorials at Maastricht University the “seven-step approach” is used, spread over three distinct phases: pre-discussion, self-study, and reporting [6]. The seven-step approach is successful, but signs of erosion are seen in the recent years. For example, using the same approach each time changes stepwise in a tick-off routine instead of stimulating and challenging students. In tutorials, important steps are being skipped. Integrating and applying new acquired knowledge is therefore considered difficult [7]. In a position paper of Maastricht University, dissatisfaction with the PBL practice was reported. Students would like to see more creativity and flexibility in the performance of PBL. There is a need for education in small groups other than only seven-step tutorial groups [8, 9]. Students of today with various backgrounds grow up in an online world and are assertive. Creative and flexible formats, a variation on the format of a regular PBL tutorial group, are therefore required [10]. Flipped classroom approaches have the ability to meet with the current students’ background, wishes, and needs and therewith are a candidate to explore design variations on the format of a regular PBL tutorial group. A main opportunity of this approach is the development of students’ deep understanding of the material [11]. A flipped classroom approach meets the following characteristics: content delivery in advance, awareness of the educator of students’ understanding, and higher-order learning during face-to-face sessions [12]. Student-centred learning activities are integrated in the classroom instead of teacher-centred instruction [13]. In a scoping review on the use of flipped classroom, results showed “much indirect evidence emerging of improved academic performance and student and staff satisfaction with the flipped approach” (12, p. 85). Also Låg and Sæle (2019) are preserved with their findings [13]. In their systematic review and meta-analysis, a small positive impact on student learning in flipped classroom formats was found. Findings stated that there is no single model for flipped classroom [12, 14]. Two elements should be implemented for a successful model, in- and outside the classroom, student learning that facilitates critical thinking, and student engagement [12]. While studies and reviews of PBL [4, 15] and flipped classrooms [11–13] are widely available, studies on the combination of flipped classroom and PBL in higher education are scarce [16].

Adding flipped classroom characteristics in an existing PBL course is with the expectation that it will further stimulate interaction, understanding and engagement of the students so that students are more eager to learn, share experiences with each other and gain knowledge. In this study, we focus on the students’ experiences of flipped classroom formats. To investigate this we adapted and investigated an existing 4-weeks course focusing on ageing in Europe at Maastricht University in which the existing PBL design has been extended with flipped classroom characteristics during several academic years. The following research question was formulated: How do first-year Bachelor European Public Health students experience flipped classroom formats? The main goals of these formats were to stimulate interaction between students and between students and teachers, to stimulate a broad understanding of the content of the course, and to gain knowledge in a way that makes them better adapted to the needs of their future working field.

## Methods

### Research Design

Underlying descriptive research was conducted in a real-life setting [15]. In this case-study, we wanted to explore design variations of PBL and flipped classroom to obtain a general impression on how students perceived these designs i.e., if they perceived that in these designs they could apply their knowledge, have their questions answered, and learnt.

### Participants

Participants were the students of the regular cohorts of the academic years, 2015-2016, 2016-2017 and 2018-2019 of the first year bachelor European Public Health Program. They followed the course “Ageing in Europe”, the final PBL course of the 2nd semester.

### Program and Course “Ageing in Europe”

The flipped classroom formats were applied in the bachelor program European Public Health at Maastricht University. This is a 3-years program (180 ECTS), including a multidisciplinary and international focus, that bridges the gap between public health science and (inter)national (especially European) public health developments and policies.

**FIGURE 1:**
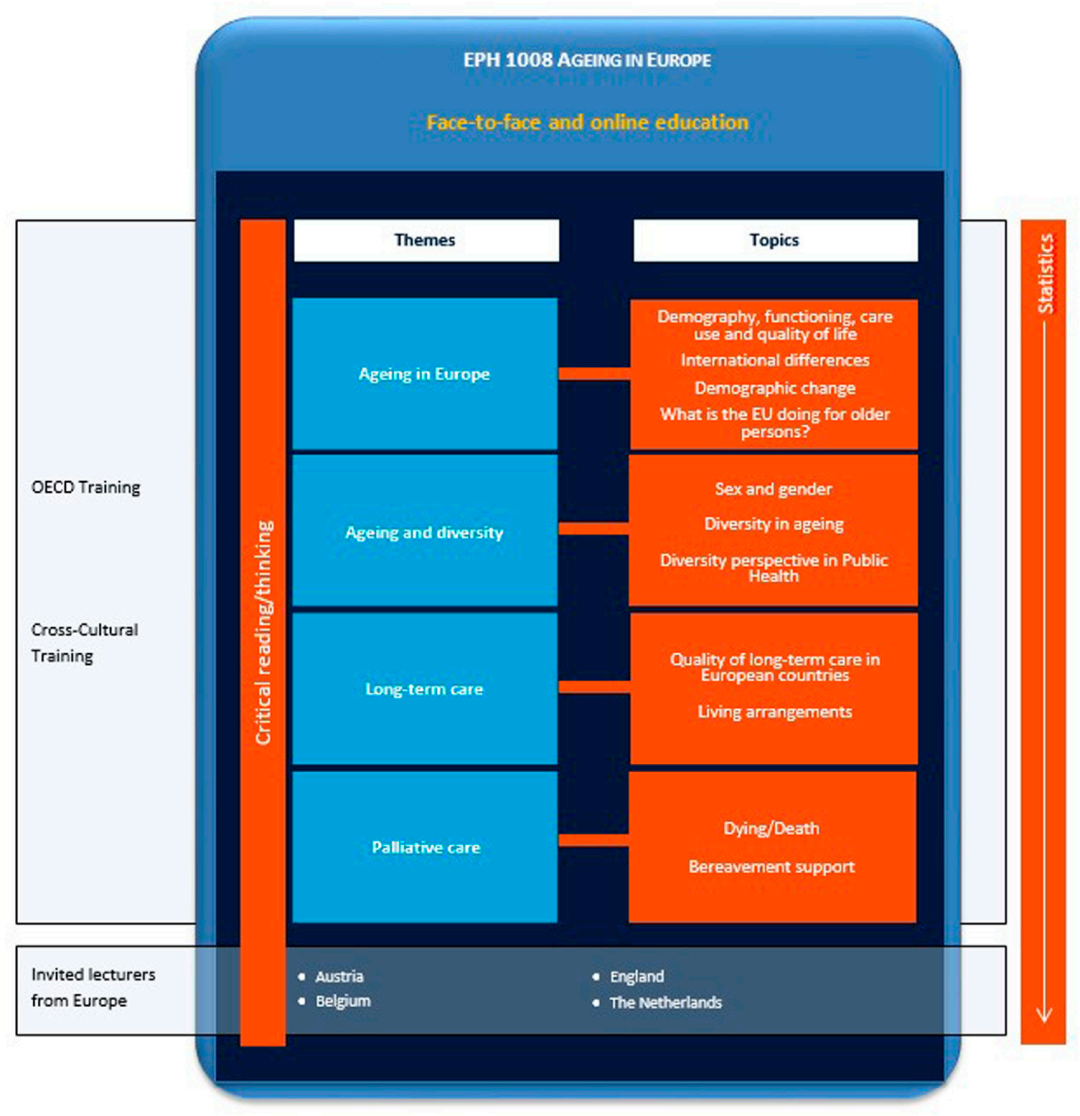
Overview of the Course “Ageing in Europe” (Maastricht 2021). Week 1 focuses on changes in the demographic profile of Europe, different views on the severity of the problem, and the solutions posited to it from an international perspective. In week 2 diversity and its relationship with inequality with respect to health and social care is the central issue of interest. Week 3 focuses on the delivery of long-term care for older people in Europe, ranging from professional home care to institutionalized care in nursing homes, and all its variations. In week 4 palliative care is addressed, including dying/death and bereavement support.

In semester 2, study year 1, fulltime students follow a 4-weeks course (5 ECTS), entitled “Ageing in Europe.” The course introduces students to the issue of an ageing European population and its consequences for health and social care. Different philosophical, ethical and policy approaches to the care of the older persons within European healthcare systems will be discussed, as well sociocultural issues. Figure 1 gives an overview of the course including the methodology, epidemiology and statistics trajectory. In three themes, ageing in Europe (week 1), ageing and diversity (week 2), and long-term care (week 3), flipped classroom characteristics were implemented.

### Educational Format: From Regular PBL Towards Flipped Classroom Formats

In Table 1, an overview of the educational format of the three study themes (“ageing in Europe,” “ageing and diversity” and “long-term care”) is presented for four academic years (2014-2015 (Y1), 2015-2016 (Y2), 2016-2017 (Y3) and 2018-2019 (Y4)). In academic year 2014-2015, the regular PBL is presented. From that academic year onwards, changes towards flipped classroom were gradually implemented. In Table 2, reasons for these changes are listed. In the paragraph below, you will read how the themes were offered in a flipped classroom format to students in the different academic years (Y2, Y3, and Y4).

**TABLE 1:** Educational format of the three themes in the different academic years (White = regular Problem-Based Learning (PBL); Black = Flipped Classroom) (Maastricht 2021).

	Academic year 2014-2015	Academic year 2015-2016	Academic year 2016-2017/2018-2019
Themes in the course (week)			
Ageing in Europe (numbers) (week 1)	In class:	In class:	In class
	1 face-to-face lecture (2 h) regular PBL task (pre-discussion)	1 face-to-face lecture (2 h) regular PBL task (pre-discussion)	1 Face-to-face lecture (1.5 h) Pre-discussion^a^ ^,^ ^b^
	Self-study:	Self-study:	Self-study:
	Reading literature	Reading literature	Reading literature
			Preparation presentation (group^b^)
			Preparation discussion (individual)
	In class:	In class:	In class^b^
	Regular PBL task (post-discussion)	Regular PBL task (post-discussion)	Presentations
			Debate
Ageing and diversity (week 2)	In class:		In class
	Regular PBL task (pre-discussion)		Pre-discussion^a^ ^,^ ^b^
	Self-study:	Self-study:	Self-study:
	Reading literature	Reading literature	Reading literature
		3 short (13–22 min) recorded lectures	Make a summary of the literature
		Follow an online module	3 short (13–22 min) recorded lectures
		Answering questions	Answering questions
			Small-scaled research
	In class:	In class:	In class:
	Regular PBL task (post-discussion)	Answering questions in small groups	Developing a mind map
	1 Face-to-face interactive lecture (2 h)	Discussion of different topics	Discussion of different topics
Long-term care (week 3)	In class:	In class:	In class:
	Regular PBL task (pre-discussion)	Regular PBL task (pre-discussion)	Regular PBL task (pre-discussion)
	Self-study:	Self-study:	Self-study:
	Reading literature	Reading literature	Reading literature
	3 short (12–20 min) video clips	3 short (12–20 min) recorded lectures	3 short (12–20 min) recorded lectures
	4 YouTube videos	4 YouTube videos	4 YouTube videos
	In class:	In class:	In class:
	Regular PBL task (post-discussion)	Post-discussion in group session	Post-discussion in group session/pitch^c^
		Role play	Role play

a Students in a tutorial groups (maximum of 12 student in each group) discuss the topics themselves; a content expert for questions is available in the same room.

b Group consists of different tutorial groups.

c The pitch was only implemented in academic year 2018-2019.

**TABLE 2:** Reasons for changes in the educational format of an academic year compare to the previous academic year (Maastricht 2021).

	Changes in 2015–2016 compared to 2014–2015^a^	Changes in 2016–2017 compared to 2015–2016^b^
Themes in the course (week)		
Ageing in Europe (numbers) (week 1)	—	Lecture is 30 min hour shorter, 1.5 h now:
		Reason: Attention of students decreased
		Remaining format is new:
		Format of the other 2 themes was successful
Ageing and diversity (week 2)	Entire format is new:	—
	Faculty member was disappointed in exam results	
	Literature was not read and discussed properly	
	The format was executed by a new faculty member	
Long-term care (week 3)	Introduction of the group session afterwards:	—
	Prepare students for future job	
	Apply knowledge	

a In academic year 2014–2015 and 2015–2016 each tutorial group had its own tutor. In academic year 2016–2017 involvement of a tutor disappeared. Experts served groups.

b No considerable changes were performed in academic year 2018–2019.

#### Theme “Ageing in Europe” (Week 1)

In all academic years the theme “Ageing in Europe” was introduced by a lecture on the first day of the course. It also acted as an introductory lecture of the course. In Y1 and Y2, the case “Ageing in Europe: a debate about numbers, facts and impact” followed a regular PBL format. In brief, this means in the first session a tutorial group (10–12 students and 1 tutor) discuss the case and set the learning goals to study. Next, in their self-study time students should study the given literature. In the second session, the students discuss and elaborate on the learning goals based on what they studied. In Y3 and Y4, the format changed. The pre-discussion in the tutorial group about the case was directed by two additional questions to stimulate discussion. However, now instead of agreeing on a set of learning goals for all to study and discuss in the second session, the students were ask to prepare for a plenary session with 3-4 tutorial groups together and present their findings to the questions and learning goals set. Afterwards, in a debate the group of students were divided into two random groups (agree group and disagree group) for discussing a statement (“In 2080 people become 130 years of age”). Before the debate, the two groups had given time for preparing themselves by exchanging their findings of literature.

#### Theme “Ageing and Diversity” (Week 2)

In Y1, a regular PBL task was offered to students. After the entire case, so after the second PBL session, an interactive lecture was given about this theme so that the lecturer made a recap to the theme and corresponding PBL task and students had the possibility to ask questions. In the following three academic years (Y2, Y3, and Y4) this format was changed. In Y2, three video clips were recorded on different topics, “Sex and gender,” “Diversity in ageing” and “Introducing a diversity perspective in Public Health.” Instead of starting with a PBL-session to discuss the case, students started with self-study and had to read the literature and, in addition, watch the videos, follow an online course and answer questions. Also, the post-discussion session was organised differently. The students did meet in a group of around 25 students. The students had to discuss the answers to the questions in small sub-groups (3–5 students) before discussing them plenary. In Y3 and Y4 the format was further adapted. The theme started with a PBL-group session (pre-discussion). Students were also asked if they had questions about the organisation of this theme. For the self-study, the video clips of Y2 were used. Literature was asked for reading as well, but making a summary of it was extra. Like the Y2-cohort, students of Y3 and Y4 had to answer questions. In addition, they had to execute a small-scale research individually. In the group session, including 3–4 tutorial groups, the diversity expert asked the students to organise in groups of four and make a mind map. The expert elaborated on the topic with each group separately. The content was also discussed plenary.

#### Theme “Long-Term Care” (Week 3)

In Y1, a regular PBL task on housing with care was offered. The self-study, however, was extended with three video clips, specifically recorded for this theme and four video clips selected from YouTube. The video clips provided explanation of the theoretical underpinnings of the impact of long-term care environments on older people as well as practice-based examples from nursing homes. In Y2, Y3, and Y4 the same PBL task was used, but the post-discussion was different. The post-discussion of the tutorial group meeting was replaced by a group session in which the learning goals were shortly discussed and the students had to prepare and perform a role-play to experience aspects of the theme. The students discussed with the experts a real-life case of Mrs. X and which housing with care option would be best in her case: living at home with support, moving into a regular nursing home or an innovative, small-scale homelike care environment. The students were grouped, each representing a specific stakeholder perspective: the older person and her daughter, the nurse and the management of the care organisation. Using the theory from the lectures and self-study the students discussed the best care option. To stimulate discussion, in Y4 student groups also prepared a 1-min pitch about an innovative nursing home within a country of their choice describing the concept and its’ underlying care principles. In this academic year, students were also able to visit a nursing home physically.

### Procedure and Data Collection

Participation in course evaluation activities was anonymously/anonymized and voluntary. Data were collected during or immediately after the course.

#### Student Perspective: Questionnaire

In Y1, no date were collected, because there was no flipped-classroom format. A questionnaire (Supplementary Appendix S1), designed by the researchers themselves, focusing on student perspectives regarding the educational format was obtained directly after the group session in Y2 (weeks 2 and 3) and Y3/Y4 (weeks 1, 2 and 3). An online survey tool, Qualtrics, was used in Y2 and Y3. In Y4, a pen and paper questionnaire was used because of results in response rates in Y2 and Y3. Fourteen closed questions focused on prior knowledge, preparation work, and group session parameters (e.g., duration, content, extent of interaction and format group session). Answers were rated on a scale from 1 (very bad) to 10 (very good), on a dichotomy scale or on 4-points Likert scale (very dissatisfied to very satisfied; fully disagree to fully agree). Students were asked to elaborate their answer in three open-end questions.

### Data Analysis

The quantitative data in the different academic years were subjected to simple descriptive analyses (frequencies, means and standard deviations). Open questions were analysed by summarizing the answers. Citations were used to enforce the findings on the closed questions.

## Results

### Participants and Response

In total 304 first-year bachelor European Public Health students followed the course “Ageing in Europe” spread over the three academic years: 2015-2016 (Y2), 2016-2017 (Y3) and 2018-2019 (Y4) with respectively, 116, 94 and 94 students. The response rate in Y2 and Y3 range runs between 15% and 64% for the questionnaires. No response rate of Y4 was calculated, because resit students could also join these sessions. In Y2 and Y3, there were no resit students in the sessions. In Table 3 students’ attendance rate of the group sessions as well as the number of students who filled out the questionnaire in Y2, Y3, and Y4 are presented.

**TABLE 3:** Rates of students’ attending the group sessions and filling out questionnaires in the different academic years (Maastricht 2021).

	Total (n)	Ageing in Europe (numbers) (week 1)		Ageing and diversity (week 2)		Long-term care (week 3)	
		Attending session	Fill out questionnaire	Attending session	Fill out questionnaire	Attending session	Fill out questionnaire
2015-2016	116	—	—	90 (78%)	62 (53%)	73 (63%)	17 (15%)
2016-2017	94	79 (84%)	60 (64%)	87 (93%)	38 (40%)	88 (94%)	30 (32%)
2018–2019^a^	94	83 (?)	76 (?)	78 (?)	81 (?)	78 (?)	73 (?)

a Students of other academic years could join when failing the exam.

### Students’ Experiences With Flipped-Classroom Characteristics

#### Experiences of Sessions in General

Students liked the in-class activities, such as debate, making mind map, giving a pitch, role-play. The interactivity and discussions with the experts during these sessions were assessed most positively. In all three academic years, students were satisfied about the duration, content, extent of interaction, and the format of all sessions.

On a range from 1 (very dissatisfied) to 4 (very satisfied) students scored an average between 3.0 and 3.4 (Table 4). Only in week 1, “Ageing in Europe,” four average score (in Y3 and Y4) were lower than 3.0. The overall grade (10-point scale) of this session was rated with a 6.5 in Y3, in Y4 this score was 7.5 (Table 4). The other two theme sessions in Y2, Y3 and Y4 scored 7.5 or higher.

**TABLE 4:** Results on how students rate the session in the different academic years (Maastricht 2021).

	*N*	Duration of session	Content of session	Extent of interaction	Format of session	Overall grades^b^			
		Mean (SD)	Mean (SD)	Mean (SD)	Mean (SD)	Min	Max	Mean (SD)	*n*
2015–2016									
Week 2 (diversity)	55	3.2 (0.60)^a^	3.2 (0.41)	3.4 (0.58)	3.2 (0.57)	3	10	7.6 (1.58)	55
Week 3 (housing)	12	3.2 (0.55)	3.0 (0.58)	3.0 (0.71)	3.0 (0.58)	5	10	7.5 (1.50)	12
2016–2017									
Week 1 (numbers)	45	3.0 (0.56)	2.8 ^c^ (0.63)	3.1 (0.66)^e^	2.7 c (0.69)	3	10	6.5 ^d^ (1.56)	45
Week 2 (diversity)	24	3.3 (0.45)	3.3 (0.52)	3.4 (0.57)	3.3 (0.61)	5	10	7.7 (1.31)	24
Week 3 (housing)	28	3.1 (0.87)	3.0 (0.76)	3.2 (0.67)	3.0 (0.73)	3	10	7.6 (1.45)	28
2018–2019									
Week 1 (numbers)	75	2.9 (0.56) ^c^ ^,^ ^f^	3.2 (0.53)	3.1 (0.56)	2.9 (0.69)	4	10	7.5 (1.16)	75
Week 2 (diversity)	78	3.1 (0.52)	3.2 (0.55)^g^	3.2 (0.68)^g^	3.2 (0.60)	3	10	7.7 (1.45)	80
Week 3 (housing)	72	3.2 (0.54)	3.2 (0.54)	3.3 (0.60)	3.2 (0.60)^h^	5	10	7.8 (1.06)	72

a Range 1 (very dissatisfied)–4 (very satisfied).

b Range 1 (very poor)–10 (excellent).

c A score below 3.0 is a point of interest.

d A score below 7.0 is a point of interest.

e *n* = 44.

f *n* = 76.

g *n* = 80.

h *n* = 73.

In Table 5, answers on three statements for the different themes are presented: “I had the feeling I could apply knowledge,” “My questions were answered” and “I have learned a lot.” For each statement, the range was 1 (very dissatisfied) up to and including 4 (very satisfied). Overall, students were satisfied. Scores were mostly rated from 2.9 and higher. Each session of the themes was recommended by 75% or more of the students, except for the session with respect to the theme “Ageing in Europe” in Y3. This session was recommended by 56% of the students.

**TABLE 5:** Results of the group sessions in the different academic years (Maastricht 2021).

	*N*	I had the feeling	My questions were answered	I have learnt a lot
		I could apply knowledge		
		Mean (SD)	Mean (SD)	Mean (SD)
2015–2016				
Week 2 (diversity)	59	3.2^a^ (0.72)	3.3 (0.73)^c^	2.9 ^b^ ^,^ ^d^ (0.74)
Week 3 (housing)	13	3.0 (0.96)	3.1 (0.62)	2.9 ^b^ (0.62)
2016–2017				
Week 1 (numbers)	48	2.9 ^b^ (0.67)	3.0 (0.58)	2.7 ^b^ (0.66)
Week 2 (diversity)	28	3.3 (0.63)	3.3 (0.54)^e^	3.0 (0.69) ^e^
Week 3 (housing)	29	3.2 (0.53)	3.1 (0.48)	2.9 ^b^ (0.64)
2018–2019				
Week 1 (numbers)	76	3.2 (0.49)	3.3 (0.49)^f^	3.1 (0.52)^g^
Week 2 (diversity)	80	3.3 (0.60)	3.3 (0.52)^h^	3.1 (0.66)^i^
Week 3 (housing)	73	3.2 (0.49)	3.3 (0.48)^j^	3.0 (0.70)^k^

a Range 1 (very dissatisfied) – 4 (very satisfied).

b A score below 3.0 is a point of interest.

c *n* = 57.

d *n* = 58.

e *n* = 27.

f *n* = 75.

g *n* = 73.

h *n* = 78.

i *n* = 76.

j *n* = 70.

k *n* = 69.

#### Theme “Ageing in Europe” (Week 1): Y3 and Y4

The majority of the students (Y3: *n* = 41, *p* = 79%; Y4: *n* = 68, *p* = 93%) reported the session fits in the course. It is not clear whether students had prior knowledge. Most students (Y3: *n* = 48, *p* = 92%; Y4: *n* = 75, *p* = 100%) prepared for the session. It is unclear how students prepared. Two students in Y3 gave an explanation for not preparing: “I didn’t feel well” and “I was busy with my student association.” More than half of the students (Y3: *n* = 29, *p* = 57%; Y4: *n* = 52, *p* = 72%) liked the format of the session. Nine students (Y3: *n* = 8; Y4: *n* = 1) liked the debate; and eleven times “discussion” was counted in a positive way (Y3: *n* = 6; Y4: *n* = 5). Also, “*we had professionals around to consult*,” “*applying knowledge*,” “*everyone participates*,” “*interactive and dynamic*,” “*it was well structured*” and “*challenging*” were filled out in the questionnaire. Eight students (Y3: *n* = 5; Y4: *n* = 3) did not like the large size of the group. 27 other students (Y3: *n* = 3; Y4: *n* = 24) mentioned the students’ presentations were too similar. Four other students (Y3: *n* = 2; Y4: *n* = 2) preferred a “normal” post-discussion.

#### Theme “Ageing and Diversity” (Week 2): Y2, Y3, and Y4

In Y2 (*n* = 57, *p* = 92%), Y3 (*n* = 35, *p* = 95%) and Y4 (*n* = 74, *p* = 97%), over 90% of the students reported the session fits in the course. More than 90% of the students did have enough prior knowledge for following the session (Y2: *n* = 60, *p* = 97%; Y3: *n* = 35, *p* = 95%; Y4: *n* = 79, *p* = 99%). In Y2, one student (*p* = 2%) did not prepare for the session. The student in Y2 gave as reason “*was not able fully prepare due to the amount of workload that needed catching up on*.” In Y3 and Y4, respectively eight (*p* = 23%) and four students (*p* = 5%) did not prepare. Two of them in Y3 gave a reason for it: “*busy with assignments of module*” and “*busy with other things*”. In Y4 one student filled out this section saying “*the last exam was last week so I still feel a little exhausted and I wanted to take it slower this week*.” How students prepare is not clear. The format of the session was liked, 150 students (Y2: *n* = 50, *p* = 82%; Y3: *n* = 28, *p* = 88%; Y4: *n* = 72, *p* = 91%)) of the 171 students in all academic years who answered the question were positive. ‘Interactive’ was explicitly mentioned as a positive aspect, also written in combination with group session and discussion such as “*interaction, space for discussion in small group*.” Next to this aspect, effective, original, size of group, productivity, structure, role of expert, possibility to ask questions and apply knowledge were also reported as positive. Eleven students (*p* = 18%) in Y2 did not like the format. Eight students gave a reason. The most mentioned reason (*n* = 5) was related to wishes in having a “normal” tutorial group meeting. Four students (*p* = 13%) in Y3 were dissatisfied with the format of the session. Three of them gave a reason: “*too big groups, no tutor per group*,” “*didn’t get in depth with the literature*,” and “*the tutor were spread finely among the small sub groups*.” Seven students (*p* = 9%) did not like the format in Y4. Reasons were, preferring a lecture, not liking the format or content and knowledge of expert was missing.

#### Theme “Long-Term Care” (Week 3): Y2, Y3 and Y4

After the group session in Y2, the expert forgot to mention to fill out the questionnaire. In Y2, thirteen students (*p* = 76%) agreed the course fits in the course. In Y3 and Y4, the majority (Y3: *n* = 27, *p* = 90%; Y4: *n* = 56, *p* = 92%) agreed on this. In all three academic years, most of the students, respectively 15 (*p* = 88%), 27 (*p* = 90%) and 67 (*p* = 99%) students, had enough prior knowledge for following the group session. The two students in Y2 who did not have enough prior knowledge gave as reason they were not prepared. It is not clear how students prepare for the group session, but most students did prepare: 13 students (*p* = 77%) in Y2, 29 students (*p* = 97%) in Y3 and 72 students (*p* = 100%) in Y4. Two out of three students in Y2, who did not prepare.

In Y2, 80% of the students (*n* = 12) liked the format of the session, in Y3 and Y4 this was, respectively, 87% (*n* = 26) and 96% (*n* = 68). Thirteen positive remarks were related to application of knowledge. Examples are: “*Real case simulation*” and “*it is close to what we are going to do in the future*.” Interaction was positively mentioned twenty times. Moreover, structure, productivity group discussion, interest, and engagement were also reported as positive aspects of this format. In addition, in Y4, twelve students were speaking highly of the pitches. Eleven students of the total group (*n* = 45) (Y2: *n* = 3, *p* = 20%; Y3: *n* = 4, *p* = 13%; Y4: *n* = 3, *p* = 4%) disliked the format. “*Role-play was not beneficial*” and “*too large group*” were mentioned as negative points.

## Discussion

The findings in this study expressed students’ perceptions of flipped-classroom formats. In-class activities, such as debate, making mind map, giving a pitch, role-play e.g., were highly appreciated by students, especially the interactivity and discussions with the experts during these sessions. Students were satisfied about the duration, content, extent of interaction, and flipped classroom formats of all sessions in the three academic years. The study found students put effort and time in preparation (prior to class) and in group sessions (in-class). R esults indicated that group sessions were highly appreciated by students, especially the interactivity of the sessions and discussions with the experts during these sessions. Students felt they applied knowledge at the end of the course. In a study of Alebrahim and Ku (2020), the majority of the fourteen undergraduate students also liked the flipped classroom format [16]. Students had to access academic media before discussing and applying the content with the professor in class. Similar to our study, their engagement was increased [16]. Following our students, the various flipped classroom formats including different preparation work and in-class activities like, debate, presentations, and role-plays e.g., did fit in the course. It is not clear which format students preferred the most. Using different flipped classroom formats can address various learning styles throughout the course, which can help students in their learning (addresses different cognitive levels) [17]. The group activities can also provide additional benefits, such as developing leadership skills, which are important for their future job [18–20]. Some of our students did mentioned that they realized the flipped classroom (the in-class part) was close to what they will do in their future jobs. T he few students who were not fond of the group sessions tend to share the same underling reasons, i.e., a too large size of the group, presentations of peers were too similar and not going into depth in literature. In order to keep the group sessions dynamic and interactive, it can be a recommendation for the future to make sure that presentations will have as little as possible repetition in content. This way, problems can be discussed from a broader perspective and eventually with even more depth. The group size of each in-class activity should be tailored made, which means group sizes can differ for various in-class activities. M ore than 90% of the students reported to have enough prior knowledge for following the session. Perhaps, profitability of the group sessions can increase with the implementation of themes of perspectives on problems, of which can be assumed that prior knowledge will be limited and peers will be more dependent of each other in order to gain this extra knowledge. As mentioned before, more focus overcomes the repetition in presentations and stimulates going into depth.

### Limitations and Future Research

Overall participation in the questionnaires (academic years 2015-2016 and 2016-2017) was fair however with a wide spread and a clear tendency to decrease each week. This can be related to students were getting tired of being asked to fill out one every week. In academic year 2015-2016, the decrease was unexpectedly large. This was caused by an omission of the expert who did forget to hand out the questionnaires after one session. In academic year 2018-2019, pen and pencil questionnaires were used to stimulate students to fill out the questionnaires. Ongoing research should also consider this. Interesting will be the fact whether the flipped classroom formats will have an effect on the motivation of students, students’ self-directed learning skills (e.g., planning, monitoring), students’ preparation for their future jobs and students’ success rates on exams. In spite of a positive finding in students’ experiences, the question is whether the regular exam still fits to the teaching and learning activities. Are the intended learning outcomes, teaching and learning activities and the assessment method still constructively aligned? Further research should explore this.

### Conclusion

Flipped classroom formats can be used to extend the Maastricht University PBL design and students do recommend this. It can be a relevant and challenging answer on the articulated request for more varied tutorial group approaches i.e., other than the regular seven-step tutorial group and on the increasing diverse public of students, with different backgrounds, studying in an environment that is no longer only based on books and written theories.
