# Supplementary material for: Flipped Classroom Formats in a Problem-Based Learning Course: Experiences of First-Year Bachelor European Public Health Students
Source: Public Health Rev. 2022 Aug 4;43:1604795. doi: 10.3389/phrs.2022.1604795 (PMC9385971; doi:10.3389/phrs.2022.1604795)
Supplement: Supplementary file 1 [file DataSheet1.docx]

**Appendix 1: Questionnaire ‘Educational format flipped classroom’**

1. Please, select an option:

|  | **Fully disagree** | **Disagree** | **Agree** | **Fully agree** |
| --- | --- | --- | --- | --- |
| **The theme meeting with EXPERT fits in the module?** |  |  |  |  |

2. Did you have enough prior knowledge for following the theme meeting with the EXPERT? **YES/NO**


3. Did you prepare for the THEME meeting with EXPERT? **YES/NO**
**If NO**, what was the reason for not preparing?


4. Statements with respect to the theme meeting with the EXPERT?

|  | **Fully disagree** | **Disagree** | **Agree** | **Fully agree** |
| --- | --- | --- | --- | --- |
| **I had the feeling I could apply knowledge** |  |  |  |  |
| **My questions were answered** |  |  |  |  |
| **I learned a lot** |  |  |  |  |
| **I could not miss this meeting because important information would have been missed.** |  |  |  |  |

5. How would you rate the theme meeting with the EXPERT?

|  | **Very dissatisfied** | **Dissatisfied** | **Satisfied** | **Very satisfied** |
| --- | --- | --- | --- | --- |
| **Duration** |  |  |  |  |
| **Content** |  |  |  |  |
| **Extent of Interaction** |  |  |  |  |
| **Format of theme meeting** |  |  |  |  |

6. Did you like the format of this theme meeting with EXPERT? **YES/NO**
**If YES**, why did you like the format of theme meeting with expert?
**If NO**, why didn’t you like the format of the theme meeting with expert?


7. What is your OVERALL grade for the theme meeting with the EXPERT? Please select an option.

**1 2 3 4 5 6 7 8 9 10**

(Very bad) (Very good)


8. Would you recommend this theme meeting with EXPERT to your fellow-students? **YES/NO**
**Comments:**
